# Catchments of general practice in different countries– a literature review

**DOI:** 10.1186/1476-072X-13-32

**Published:** 2014-08-29

**Authors:** Donald P Allan

**Affiliations:** 1 Discipline of Public Health, School of Health Sciences, Faculty of Medicine, Nursing & Health Sciences, Flinders University, Health Sciences Building, Registry Road, Bedford Park, SA 5042, Australia

**Keywords:** General practice catchment areas, Accessibility measures, GIS, Spatial scale, Markets, Primary health care, Patient registration catchments

## Abstract

The purpose of this paper is to review the current research on catchment areas of private general practices in different developed countries because healthcare reform, including primary health care, has featured prominently as an important political issue in a number of developed countries. The debates around health reform have had a significant health geographic focus.

Conceptually, GP catchments describe the distribution, composition and profile of patients who access a general practitioner or a general practice (i.e. a site or facility comprising one or more general practitioners). Therefore, GP catchments provide important information into the geographic variation of access rates, utilisation of services and health outcomes by all of the population or different population groups in a defined area or aggregated area.

This review highlights a wide range of diversity in the literature as to how GP catchments can be described, the indicators and measures used to frame the scale of catchments. Patient access to general practice health care services should be considered from a range of locational concepts, and not necessarily constrained by their place of residence. An analysis of catchment patterns of general practitioners should be considered as dynamic and multi-perspective. Geographic information systems provide opportunities to contribute valuable methodologies to study these relationships. However, researchers acknowledge that a conceptual framework for the analysis of GP catchments requires access to real world data. Recent studies have shown promising developments in the use of real world data, especially from studies in the UK.

Understanding the catchment profiles of individual GP surgeries is important if governments are serious about patient choice being a key part of proposed primary health reforms. Future health planning should incorporate models of GP catchments as planning tools, at the micro level as well as the macro level, to assist policies on the allocation of resources so that opportunities for good health outcomes for all groups within society, especially those who have been systematically denied equitable access, are maximised.

## Introduction

### Primary health care as an important issue in different countries

Healthcare reform, including primary health care, has featured prominently as an important political issue in a number of developed countries. For example, in the recent USA presidential elections, the debate around the Patient Protection and Affordable Care Act (PPACA), also known as Obamacare, was intense [1]. In the United Kingdom, ongoing debate in the UK National Health Service (NHS) centres on the role of general practitioners (GPs) not only as providers of primary care, but as procurers of secondary (hospital) care [2]. In Australia, reform of the primary health care sector, including the introduction of Medicare Locals, was a key strategy in the Labor Government’s reform program, which has been subsequently shelved by the incoming Liberal Government [3,4]. In Switzerland, in June 2012, the Swiss voted against a new health initiative by the Government to control cost inflation, and address the challenges posed by population aging, chronic disease, and the cost of new technology [5].

The debates around health reform have had a significant health geography focus. For example, in the USA, the prestigious Institute of Medicine on behalf of the Affordable Care Act, published a report entitled “Variation in Health Care Spending: Target Decision Making, Not Geography” [6]. This report was perceived by a number of commentators as criticizing the work of the Dartmouth Institute for Health Policy and Clinical Review, which had advocated, through its Dartmouth Atlas of Healthcare, over many years that geography is important and that a geographic focus on health reform is strong [7,8]. In the UK, it is claimed that reforms envisaged by the Conservative Government, if implemented will have profound geographic implications on primary care [9]. In Switzerland, it was argued that the proposed reforms would create a two tier system with regard to general practice choice [5]. Within these debates, equity of access to primary health care services is an important issue [10]. General practice is considered an important part of the primary health care [11] and is more often considered to denote the first contact, accessibility, continuity and permanence of care provided in association with other sectors [12].

### The purpose of this review

In the context of policy issues and debates around health reform, and specifically maximising access to primary health care, the purpose of this paper is to review the literature on private sector GP catchments in different countries over the past twenty five years. Conceptually, GP catchments describe the distribution, composition and profile of patients who access a general practitioner or a general practice (i.e. a site or facility comprising more than one general practitioner). Therefore the composition of GP catchments provide important information into the geographic variation of access rates, utilisation of services and health outcomes by all of the population or different population groups in defined local areas, or aggregated areas at higher spatial scale. Conceptual models of GP catchments, based on real empirical data, therefore have the potential as planning tools to assist health planners identify where opportunities for good outcomes have been systematically denied to groups within society and where access issues need to be addressed.

### Background to the review

The background to this review originated from the development of case studies, using empirical data, of the catchments of private general practices in Australia, based on the model of the Practice Health Atlas [13]. Through the Australian Divisions of General Network, staff at the Adelaide Western Division of General Practice trained the staff in over 50 Divisions in the use of geographic information systems associated with the Practice Health Atlas method. It is estimated that more than 500 case studies of individual general practices were produced through the Divisions. Unfortunately, to date, peer reviewed articles based on this important source of empirical data on the nature and composition of catchments of private general practices, including information from where patients access their general practice, have not been available.

### General practice catchments and primary health care policy in different countries

The potential value of general practice catchments as a method to be used in small area health care studies, as a contribution to informed primary health care policy, prompted the development of this review of the international literature. The WHO defined the concept of primary health care as a strategy and a set of activities to reach the goal of “health for all by the year 2000” [14], however, since the Declaration, different schools of thought, and even confusion, as to the differences between selective primary health care, comprehensive primary health care and primary care, have evolved [15]. As Crooks and Andrews note, many governments have since created their own working definitions of primary health care [16]. The central attributes of primary health care, according to a literature review conducted by Maeseneer et al. in 2007 [17], are: first contact (accessibility), longitudinally (person-focused preventative and curative over time), patient-oriented comprehensiveness and coordination.

Primary health care can be provided by a range of health providers, including general practitioners (or physicians as they are referred to in the US, Canada, and often in the international literature), dieticians, nurses, nurse practitioners, occupational therapists, psychologists, physiotherapists, pharmacists, social workers, and other health care providers [18]. In this review, the term general practitioner and general practice are used throughout and include other similar used terms in different countries such as physician (not specialist physician as in the US), family physician and doctor. Further, the review narrowed and limited its focus to general practice (as one set of primary health care providers), and the attribute of accessibility, as one of the key attributes of primary health care because these concepts provided the central framework for the review of catchments of general practice.

### Catchments and access

In the context of the review, the term catchment is assumed to mean a geographical area around the general practice that includes the client population which accesses its services [19]. At the outset of the review, no limitation was placed on the scale of the catchment, and therefore the scale might range from the local to the national, provided that there was a clear link between the provider of the service and the client population accessing the service.

There are many ways of thinking about access to primary health care. Crooks and Andrews [20] have noted that access is a topic of great interest to health geographers, and whilst geographic access to care is clearly overtly geographic, health geographers interest in access extends beyond this. They note that certain health professionals (e.g. general practitioners) serve as gatekeepers to people’s access to secondary and tertiary care and given their centrality to primary health care in many countries, their practice (both spatial and non-spatial) plays an important role in people’s access to primary health care.

A review of the literature on access to primary health care was conducted by Ansari [21] in 2007 who observed that whilst policy-makers are concerned about providing equal access to health care for all, there was a significant lack of detail in the plethora of policy documents regarding what is meant by the term “access”. In the review, Ansari [22] found that the concept of access to health care has long been ill-defined, with no clear consensus on its definition in the literature and no universally accepted way to measure it. Ansari summarised the theoretical work of various researchers into a framework of access . The model of Penchansky and Thomas [23] is referred to by Ansari as a general model of access covering five dimensions of access which include availability, accessibility, accommodation, affordability and acceptability. Ansari then refers to the work of Donabedien [24] and Khan and Bhardwaj [25] who further sub-divide the accessibility dimension into two sub-categories, termed socio-organisational and geographic (Donabedian) and aspatial and spatial (Khan and Bhardwaj).

Recent writers have provided further perspectives on accessibility, spatial access and spatial equity of access in primary health care. For example, Wang [26] argues that spatial access emphases the importance of spatial separation between supply (i.e. health care providers) and demand (i.e. the population) and how they are connected in space and thus is a classic issue for location analysis. Wang suggests that in the USA, the main debate revolves around what are reasonable catchment areas (sizes) for general practices. As noted in the introduction, in the US, the Patient Protection and Affordable Care Act (known popularly as Obamacare) has generated controversy over measures to assess access to general practitioners. For example, the Manhattan Institute for Policy Research [27] has initiated an ongoing evaluation of the PPACA with an evaluation of the law’s effect into five areas including access to care. The researchers noted that geographic maldistribution of health care resources is an important issue, but chose not to investigate it. Instead, they acknowledged that the issue of geographic maldistribution of health care resources has generated different explanations from variations in Medicare spending to differences in treatment intensity [28].

In the UK, Lewis and Longley [2] focus on the spatial equity of access to health systems, and this includes the question of who benefits and why in the provision of urban services and facilities. Lewis and Longley note that the proposed reforms in the UK, in their present state, would seek to abolish catchment areas.

In summary, the focus of this review paper therefore has a quite specific focus on the role of catchments of private sector general practitioners within primary health care. Catchments provide an area of study where the effects of government spending (such as the spending arising from the new Obamacare Law) and geographical variation in access to GP services can be studied together. A review into the literature on GP catchments in different countries is important because it should provide important perspectives on the nature of health care accessibility and therefore inform public policy debates on primary health care health care reform.

### The aims of the review

Research questions on the review of the literature on catchments were formulated to incorporate the perspectives as outlined above.

This review aims to examine the existing literature in order to find out:

• How are GP catchments described and in what context?

• What indicators and measures are used in the description of catchments?

• What relationships between the health seeking behaviour of patients and GP catchments are studied in different countries?

• What relationships between the concept of a GP catchment and public health policy (including regulation) issues are studied in different countries?

The outcome of the review will include identifying areas where there are the gaps in the literature and suggesting areas of research worth further investigation.

## Method

### Overview

The goals of the literature review were to: (1) conduct a literature review of peer reviewed papers (titles or abstracts) with any mention of GP catchments in relation to primary health care; (2) identify from title or abstract, key words in relation to the term GP catchment that could facilitate further narrowing of the search; (3) to synthesise the articles into a number of broad themes in relation to the aims of the review.

### Search strategy and selection criteria

The search strategy was conducted in four stages. The first stage was a pilot stage, with a search of the database of the International Journal of Health Geographics (IJHG), which is a journal known to publish many peer reviewed articles within the theoretical framework. Using the key words “catchments in health”, this search yielded 21 results. The search also included two previous literature reviews and their citation lists. One paper [29] had a primary focus on the methodologies used to examine access through Geographic Information Systems (GIS) approaches and the second [30] examined concepts, methods and challenges in spatial accessibility. An iterative approach involving key words identified in the literature reviews and in the peer reviewed papers from the initial search, was adopted to expand the search in the database of the IJHG and in the database of another journal, Social Science and Medicine. This search yielded a further 182 results.

This iterative process identified key words as relevant to the literature on catchments of general practice in primary health care. The key words included: Geographic Information Systems (GIS), catchments and markets in health, competition in primary health care markets, the Two Step Floating Catchment Area (2SFCA) method, spatial accessibility and primary health care; GIS and primary health. In the search, the term physician was recognised as meaning the same as the term general practitioner and it was therefore also included in the search.

The second stage of the search strategy was conducted through MEDLINE, using the databases of Web of Knowledge and PubMed. Google Scholar was used after the other searches were complete. The initial search began with the Web of Knowledge database using the key words: general practitioners and accessibility, covering all years and in the English language. This yielded 1,321 results and then further filtering was undertaken. The filters included:

General practitioners and accessibility and catchments (N = 42 results);

General practitioners and accessibility and spatial accessibility (N = 14 results);

General practitioners and accessibility and GIS (N = 14 results);

General practitioners and GIS (N = 35 results);

Accessibility and catchments in primary health (N = 156 results);

Access and physician market areas (N = 95 results);

General practitioners and accessibility and markets (N = 21 results).

The process was then repeated with PubMed, using the key words: general practitioners and accessibility in order to cross reference the search in the Web of Knowledge. The search yielded 908 results. A similar filtering process was then undertaken. In PubMed, the category of the Two Step Floating Catchment Area method was searched and this yielded 101 results. Total results prior to exclusion were 1,422 results.

The process of exclusion included eliminating articles by title or abstract if the article did not relate in any way to private sector general practice, general practitioners or physicians]. Peer review papers were selected and prioritised based on their inclusion of two or more of the key words in abstract: accessibility in primary health care, the concept of catchments or markets; private general practice; and preferably GIS concepts, methodology or terms. Prioritisation also included an assessment as to how the article rated overall to the search criteria. This meant that peer reviewed papers covering accessibility and one or more of the other access dimensions (e.g. the availability dimension) could be eligible for inclusion.

**Figure 1:**
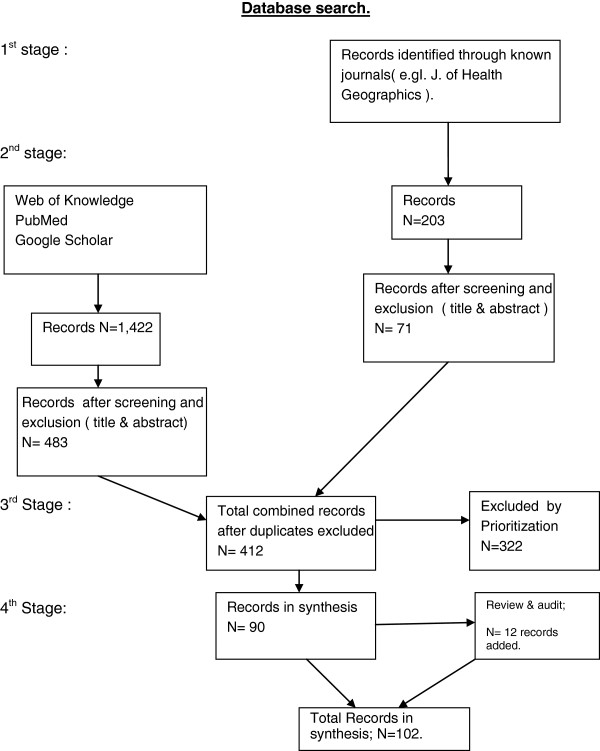
Search process and results.

The third stage of the search process involved a quality audit, based on feedback received from independent reviewers. This process identified a number of relevant papers, where the term “general practice” or variation was not contained in the title, but where the context of the paper related substantially to one or more of the aims of this paper. This process identified another 12 papers for inclusion. A total of 102 peer reviewed papers were identified as relevant to the review. The search process is shown in Figure 1. The fourth stage involved an analysis of the title and abstract of each of the papers based on the criteria as shown in Table 1. The criteria for Table 1 was developed in the context of the aims of the review. The results of the analysis are also shown in Table 1.

**Table 1:** Summary of results: the analysis of the studies in the review in relation to GP catchments

**Criteria**	**Category**	**Number**	**Total number in the category**	**Percentage of total studies**
What is the major theme in the article as outlined in the title?:	Literature review	4	102.	100%
	Measurement as the key theme in the research study.	16.		
	Spatial access as the key theme	30.		
	The role of markets, and competition between catchments of general practice, in primary health care.	23		
	The Neighbourhood as the focus of the research inquiry.	10.		
	The role of patients choice as the focus of the study.	9		
	Studies in relation to access issues in primary health care.	6.		
	Other issues of methods and scale.	4.		
What Spatial scales are applied in the article?	Neighbourhood.	9	75	73.5%
	Region/metropolitan	36		
	Rural	10		
	State/National	10		
	Multi-scale	10		
What Indicators and measures & data sources are used ?	Socio-economic/demographic/Ethnicity/Census data./population cohorts at regional, state, national (inc postcode data)	31	75	73.5%
	Patient registrations./lists/retail clinics	9		
	Multiple/various (Including Medicare data, other health care data.	20		
	Observational studies/cohorts/surveys	5		
	Other large data sets – GP workforce data sets	10		
What are the GIS methods, if any, applied in the paper	2 SFCA method	26	43	42.1%
	Other methods of GIS (including those referenced in Table 2)	19		
What specific Population cohorts, if any, were used in the study?	Elderly	2	32	31.4%
	Ethnic groups	2		
	Rural	7		
	Immigrants.	4		
	Urban/Neighbourhoods	14		
	People with disabilities	1		
	Out of hours groups	2		
What Access issues/Primary health care were referred to in the paper? (including non-spatial factors)	Reform/politics	14	77	75.5%
	Workforce	10		
	Spatial equity/Accessibility/Access to care	20		
	Patient choice/consumers/registration	7		
	Health inequalities	12		
	Socio-organisational/Practice characteristics	7		
	Access to retail clinics	5		
What was the country of origin of study	USA	35	102.	100%
	Canada	6		
	UK	17		
	France	5		
	Switzerland	3		
	Germany	2		
	Singapore	3		
	Australia	11		
	New Zealand	4		
	Europe (several countries in same paper)	8		
	Not specified	8		

(Analysis by: title, abstract and key words).

## Results

### Overview of the results of the search

Table 1 shows the analysis of the journal articles in relation to the search criteria and the aims of the search. Using the title, abstract and key words, the articles were analysed by a number of categories consistent with the aims of the review. The analysis formed the basis of reviewing the articles in the context of the aims and the results are presented in the following sections.

There were 102 papers within the study. There were four literature reviews, one from 2004, one from 2005, one from 2007, and one from 2012. The papers were sourced from 10 different developed countries. There were a range of population groups referred to in the papers, ranging from non-specific neighbourhood or regional populations to specific disease population groups, or particular service needs groups, such as immigrants or out of normal hours population groups. Studies ranged from a spatial scale on the neighbourhood through to state and national. There were five different types or variation of catchment models identified, but the Two Step Floating Catchment Area model was the model most frequently used in the studies. Patient choice was identified as a common theme in the studies that related to the health seeking behaviour of patients in terms of access to general practitioners. Studies that explored the relationship of GP catchments and the policy framework of the state or national government illustrated the concerns that government intervention in the primary health care market would affect the economic decision making of private sector GPs, and their decisions about the scope and supply of their services to consumers.

### The results

#### Studies of GP catchments in relation to their description and context

##### Overview

In relation to descriptions of GP catchments, Table 1 shows that relatively few of the articles in the review contained the words “GP catchment” in their title. The search had to analyse the articles in the details of the abstracts and even the text of the articles to determine the context in which GP catchments were discussed. This lack of common framing of the term GP catchment in the journals has lead to the development of at least five different catchment models (Table 2). Four of these models have been based on empirical data and have attempted to describe what an actual GP catchment or group of catchments within a region might look like. In addition, a wide range of spatial scales, indicators, measures and data sources are used in conjunction with studies in relation to GP catchments (Table 1).

**Table 2:** Examples of catchment models of general practice in different countries

**Model**	**Definition**	**Health example**
Two Step Floating Catchment Area method. (and enhanced 2SFCA method) (2000, 2003, 2009) USA.	A special case of the gravity model, using a special form of the physician to population ratio. The enhanced 2SFCA method addresses the problem of uniform access within the catchment by applying weights to different travel time zones to account for distance decay.	It measures spatial accessibility to primary care physicians.
		It reveals spatial accessibility patterns more consistent with intuition, and delineates explicit health professional shortage areas.
Synthetic Data Matrix (SDM, 2005) Northern Ireland data.	Compares patient to GP flow (affiliation) information aggregated at the Census Enumeration District level across a number of catchment areas using different methodologies. The SDM is then analysed using a modified version of the European Regionalisation Algorithm to create an optimal set of non overlapping regions according to predefined population size and self-containment criteria.	General practices within a defined health region.
Practice Health Atlas method. (PHA, 2006, 2010) Australia.	Provides catchment maps of patients of individual general practices, based on post code place of residence. Maps patient catchments in 7 chronic disease categories. Details level of utilisation of health services and documents level of health outcomes for specific disease groups. Describes the general practice market share of each individual postcode within a region.	General practices within a defined health region. The PHA has the capability to measure individual catchments or aggregated catchments at city or regional level.
Local Potential Accessibility (LPA) measure. (2012) France.	The LPA indicator measures the supply and demand for general practice services by taking into account practitioners’ volume of activity on the one hand, and service use rates differentiated by population age structure on the other.	The LPA indicator to private GPs indicator reveals a greater degree of variability than the traditionally used accessibility indicators (travel time, level of GP density in living areas …)
Lewis & Longley Model (2012) England.	An analysis of a data set, derived from the National Health Service Central Register. The Model compares the observed patient registrations at GP surgeries with an optimum geographic pattern.	From the London borough of Southwark. The has a focus on spatial equity of access.
	The Model also uses a new ethnicity classification tool to assess the ethnic dimensions to deviations from the normative arrangement.	The Model maps the role of the GP surgery as a place that provides local services in away that tries to serve the population as a whole (spatial equity).

##### Theory

From a theoretical perspective, two articles [31,32] reflect on the role of Central Place Theory in urban and rural planning. In Australia, Smailes argues that individuals can belong to a number of social catchments at different scales and the degrees of attachment people feel to various scales of attachments is an area of needed research. Hugo, Smailes et al. [31] define social catchments as the territory occupied by a group of households and individuals who are in some form of regular interaction and which the inhabitants identify as their community of interest. They question the meaningfulness of administrative boundaries for planning purposes and instead advocate research to develop a nested hierarchy of social catchments for social, economic and community planning in non-metropolitan areas. Hugo, Smailes et al. used the catchments of general practice in rural Australia to illustrate their arguments. Since 2000, there seems to have been a lack of theory application in regard to studies of catchments. For example, as Root notes [33], huge bodies of geographic theory on the modifiable areal unit problem (MAUP) and the relevance of scale have all but been ignored.

##### Examples of GP catchment models

Table 2 outlines examples of catchment models as developed in different countries. Each model has been developed in the context of the primary health care policy of the different country and the role that GPs fulfil in providing primary health care services within the overall policy framework.

In North America, as Shortt et al. note [34], the free market economy dominates and this results in greater patient choice and increased competiveness between health services. However, previous methodologies have been based on the importance of competing health care institutions in the secondary care market, at the expense of the primary care sector. Data on private sector GP catchments is thus considered as commercial –in-confidence and highly valuable. Models in the US have been developed using either Medicare, Medicaid data or related public health data, or aggregate data about the numbers of GPs and the population in defined areas as in the case of the Two Step Floating catchment Area model. Specific examples of private sector GP catchments or groups of catchments could not be identified.

In Northern Ireland, Shortt et al. [35] reported there was a lack of sophistication in the definition of medical service areas and that a new methodology needed to be developed. Shortt identified that GP catchment areas, though an essential basis for providing GPs with important information such as levels of accessibility to surgery, were rarely or clearly accurately defined. Previous definitions of GP catchments had been defined to single regionalisation methods, such as mean distance measures, which were prone to overestimation or underestimating medical service areas.

To address these shortcomings in the description of GP catchments in Northern Ireland, Shortt et al. developed a technique involving the creation of a Synthetic Data matrix (SDM) which compared patient to GP flow (affiliation) information aggregated at the Census Enumeration District level across a number of catchment areas using different methodologies. The SDM was then analysed using a modified version of the European Regionalisation Algorithm to create an optimal set of non-overlapping regions according to pre-defined population size and self containment criteria. In policy terms, as Shortt argues, the benefit of optimal catchments lies in their discrete nature (in terms of boundaries) and their applicability for detailed locality planning and resource allocation [35].

In England, Lewis and Longley [2] developed an analysis of a previously unexploited data set comparing the observed pattern of patient registrations at GP surgeries with an optimum geographic pattern in the London borough of Southwark. The research by Lewis and Longley represents an important breakthrough in the development of a conceptual framework of GP catchments because they were able to map the actual percentage of patients using the nearest GP surgery. In addition, through their analysis, they were able to profile the population characteristics within the catchment. From this analysis, they were able to identify the role of the GP surgery as a place of importance as it provides local services in a way that tries to serve the population as a whole (i.e. spatial equity). Longley and Lewis argue that understanding the catchment profile of individual GP surgeries is important if the National Health Service (NHS) is serious in its mantra about patient choice being a key part of its proposed reforms.

In Australia, the development of the Practice Health Atlas [13] offered some similar potential. The Practice Health Atlas (PHA) requires access to real patient data of the general practitioner, de-identified, to provide a catchment map of the percentage of patients, at the post code level. The PHA maps patient catchments in 7 chronic disease categories and it provides details of the level of utilisation of health services and level of health outcomes for specific disease groups. The PHA identifies the general practice market share of each individual postcode within a region. It includes a service substitution model which details the optimal level of services that a general practice could be providing to its patients; ie. the gap between actual services and potential. The PHA also models how the practice can substitute services from a general practitioner to a nurse, thereby enhancing the capacity of the practice to increase its utilisation of services for patients. The advantage of this data level analysis is that it removes the need to make theoretical assumptions of patient travel time of the general practice. It also provides strong data on consumer preference, such as the percentage of patients who might be accessing the practice based on their work location rather than residential address. The Practice Health Atlas was adopted extensively by Divisions of General Practice in Australia to assist general practices. Since 2011, Divisions have been replaced by Medicare Locals, and very little research using PHA data has been published in the peer reviewed literature.

In France, the Local Potential Accessibility [36] measure, offers alternative ways of assessing general practitioner provider to patient population accessibility measures. The Local Potential Accessibility (LPA) requires access to general practitioners’ volume of activity on the one hand, and service use rates differentiated by population age structure on the other. As Bartlet et al. explain, the new indicator permits reviewing data concerning the differences in medical services density and access to care between urban and rural areas. Traditionally, as they explain, spatial accessibility to GPs has used density of GPs and travel distance to the nearest GP. However, this does not take into account the number of accessible GPs. The density indicator provides aggregated healthcare supply available in a given area, but has the inconvenience of not taking into account interactions with neighbouring geographical areas. GP density thus ignores population movements across administrative boundaries, even though they are frequent for small geographic areas. The LPA attempts to offset these limitations by calculating an indicator for each municipality taking into account supply and demand within the municipality and neighbouring municipalities.

Recent articles [37-40] continue to question methodologies based on administrative units. The articles advocate the need to reconceptualise the concept of place based perspectives in general practitioner health services and invoke the need to explore the theoretical contributions of time geography and spatial ecology as opportunities to integrate human agency into contextual models of health.

In summary, more journal articles that produce examples of actual GP catchment profiles at either the individual, local, or group level are required so that a conceptual framework of GP catchments, as they are currently configured in different countries, can be established. Without practical examples of GP catchments, then the debate on health reform and how to allocate resources remains largely controlled by those groups or vested interests who benefit from the current status quo, and such a situation is unlikely to address issues of spatial inequality to health care.

#### Studies of GP catchment areas and related indicators, measures and models

From Table 1, it can be seen that studies in relation to GP catchments ranged in spatial scale. A nomenclature, such as a neighbourhood, local area, region, an administrative unit, out-of-hours area or a specific disease population group, is often used to describe such an area. They are place based studies.

##### Neighbourhoods

There were twenty nine articles [33,34,36-61], of which the vast majority were published since 2004, where the focus of the general practice primary health care study related to geographic unit of size. Of these, fifteen [33,36-38,41-51] had a specific focus on primary health and the neighbourhood as the unit of size. Within the fifteen articles, the themes included inequality, ethnicity and cultural diversity, socio-economic factors, rural health, the built environment, the role of spatial and aspatial (social) factors and even local traffic conditions. A range of methodologies of analysis were used, including GIS (Two Step Floating Catchment Area method), individual in-depth interviews, a community resilience model incorporating spatial analytic strategies, asymmetric spatial modelling, and social modelling of cultural groups.

The unit of measurement to define neighbourhoods also varied from administrative units (such as collection districts requiring a geographic spatial scale) to the “inner characteristics” of a neighbourhood or the neighbourhoods’ idiosyncrasy [48]. Lebel et al. make the observation that the neighbourhood integrates place as well as people and therefore its conceptualisation must consider characteristics of both place and people, and the interaction between them. It must also consider that a neighbourhood is always part of a larger area [48]. A multi-dimension concept of neighbourhoods is recommended by Lebel in the study of health inequalities. Lebel argues that the method could be used in urban and rural areas, leading to a possible new perspective on the nature of neighbourhoods in rural areas. Natural neighbourhoods as compared to administrative or census tracts, using a variation of the Two Step Floating Catchment Method was also reported by Bissonnette et al. [44] with implications for identifying intra-variations in access to health care. McGrail and Humphreys [58], on the other hand, developed a new index of access to rural primary care services using the Two Step Floating Catchment Area method and service health data using collection districts. Odoi et al. [49] studied neighbourhoods, which they defined as being the same as census tracts, and they used multivariate techniques (principal component analysis – PCA) to characterise neighbourhoods as units of analyses in investigating equity in health status, access and utilisation of health services. Odoi argues that a “one-size-fits all” planning approach is neither efficient nor practical and that planning strategies based on single variable analysis may not be appropriate since the strategies would not be tailored to the unique characteristics and needs of neighbourhood types.

##### Catchments and specific populations

There were seven articles reviewed, of which five of the articles [52,54-57] identified issues of spatial inequality for cultural minorities at the neighbourhood level in accessing physician health care. The studies incorporated the Two Step Floating Catchment Area method along with other methods, such as questionnaire surveys or probability studies. There were two articles [60,61] on out of hours care and patient populations. The method of analysis included survey data in one article [60], but the use of patient call out service data in the other. The analysis of catchment areas varied with one [61] using an area level index to measure rurality and in the other, there was an absence of strictly defined catchments areas. In the first study, differences were in service usage identified due to the factors of deprivation and distance (including differences between rural and urban areas), and in the second, higher representation of certain socio-economic groups, but this could have been due to these patients coming from broader catchment areas.

In summary, this section has outlined the wide range of ways in which GP catchments have been incorporated into the literature. However, the diversity of descriptions and use of a wide of indicators may not be helpful in clarifying the issues in the political debate over the allocation of resources to primary health care in periods of fiscal restraint. Greater uniformity and use of common terms, based on empirical studies, would assist the development of political arguments to advocate on behalf of those in need.

#### Indicators and measures using geographic information systems (GIS) methodology

Developments in geographic information systems (GIS) methodology have enabled a surge in the number of studies exploring the distribution of, and accessibility of health service providers to primary care populations. Two previous literature reviews, Guagliardo [30] in 2004 and Higgs [29] in 2005, have described and explained key issues, concepts and measurements of spatial accessibility of primary care. A further methodological review on measurement, optimization and impact of health care accessibility was published by Wang [26] in 2012.

The three reviews recognise that access to healthcare has multiple definitions. Guagliardo argues that this has lead to confusion in distinguishing between the ability to get care, the act of seeking care, the actual delivery of care and related indicators. The articles cite various definitions of access, but judge potential accessibility and revealed accessibility as key issues. Each article recognises a number of barriers which can impede progression from potential to realised access. These barriers, first identified by Penchansky and Thomas [23], include: availability, accessibility, affordability, acceptability and accommodation. Availability and accessibility are spatial in nature and this is where studies using GIS have focussed. Guagliardo coins the term spatial accessibility for such studies and identified most published measures of spatial accessibility to health in four categories: provider to population ratio; distance to nearest provider; average distance to a set of providers; gravitational models of provider influence.

In describing recent developments in the use of GIS methodology, each of the reviews [26,29,30] single out the Two Step Floating Catchment Area (2SFCA) method as an important new development as a method of measuring GP to population ratios, and identifying areas of GP workforce shortage. The 2SFCA method is described and critiqued in the articles. Higgs [62] describes the 2SFCA method as a relatively sophisticated technique that accounts for interactions between patients and general practitioners across administrative boundaries by evaluating accessibility as the ratio between supply and demand, both of which are determined within travel time catchments. In the first step, as described by Higgs, a travel time catchment is computed around each supply point (e.g. a primary healthcare practice) and its estimated population count utilised to calculate a GP to population ratio. In the second step, travel time catchments are computed around each demand centre (e.g. a population weighted centroid) and accessibility to service provision is measured by summing all general practitioner to population ratio values contained in the zone. The final accessibility measure reports the balance between doctor availability (ie general practitioner to population ratio) and service accessibility (sum of all supply points lying within a given travel time of the demand centres), returning higher values as accessibility increases.

Since the enhanced 2SFCA method was developed by Luo and Wang, the number of studies [26,50,55-58,63-82] using the methodology, or referring to the methodology, is more than any other specific GIS methodology. In this review twenty six studies were identified since 2004 that have applied the 2SFCA method to measure spatial accessibility. Wang in his 2012 review provides a comprehensive coverage of the papers, and includes other papers, notably in the USA, that have explored and fine tuned the 2SFCA method [26]. A common theme in the studies included the application of the 2SFCA method to measure spatial accessibility and to identify areas with a shortage of general practitioners. Some studies [50,55-58,65-68,71] combined other factors such as socio-economic factors or ethnicity to the 2SFCA to identify specific low areas of access to general practitioners. Studies included areas within metropolitan areas, a region, a state or even a nation state.

Limitations in the strength of the 2SFCA method are acknowledged by reviewers and even advocates of the method. For example, as Guagliardo notes [83], it is intuitive that communities located at insurmountable distances from any source of healthcare will be negatively impacted by the lack of resources. Both Guagliardo [83] and Higgs [62] note that critics have commented that daily activity spaces, such as where patients work, may be more representative of an individual’s location than residential address. All three reviews recognise that a key limitation in modelling is the lack of available empirical data on “real” health service access behaviour and its relationship to geography. The 2SFCA method, as McGrail [63] notes, requires assumptions of catchment size, distance-decay and the variable application of the variable application of these across metropolitan and rural populations, but to date, many applications of the 2SFCA method have not been verified against empirical access behaviour data. Wang [26] notes that the increasing complexity of the accessibility models hinder their implementation and adoption by health professionals. Wang also notes [26], the main debate centres on what are reasonable catchment area sizes for general practitioner services, but that the debate as to what is the right size for catchment areas cannot be settled without real world data. Other studies [75,76,81,82,84-86] have explored a range of measures using GIS methods at the regional or larger scale to develop stronger evidence based methods to assist in health planning.

In the US, Root [33] has been also critical of geographic information systems tools, arguing that the analysis has not been particularly sophisticated, and that huge bodies of geographic theory on the modifiable areal unit problem (MAUP) and the relevance of scale have all but been ignored. However, Taylor et al. [87] argue that the use of GIS as a tool to determine where clinics can be placed to maximise access to care is particularly relevant for primary care services associated with ongoing changes to the health system, especially in light of the Patient Protection and Affordable Care Act 2010. The study by Taylor et al is important because it demonstrates a way in which GIS can be used for administrative decision-making at the local public health level. Using empirical data, the researchers were able to model primary care centre locations and patient residences within a six mile catchment. This enabled the decision-makers to consolidate the number of centres with no or minimal changes to patient travel distances. One of the limitations acknowledged by the researchers was that using patient residences may be a limitation given that some patients travel from their workplaces or schools to the nearest primary care centre.

In summary, the use of geographic information systems has developed significantly over the past ten years, but there remains considerable scope for the use of GIS to critically analyse and model the scale and composition of GP catchments in the future.

#### Studies of GP catchments and the health seeking behaviour of patients

In relation to the third aim of the review, consumer choice and preference in the selection of primary care general practitioner is explored. Nine articles [88-96] focussed on how and why consumers either changed general practitioners or by-passed local primary care general practitioners. In the US, one study [88] found that overall 32% of respondents bypassed local primary care. A common theme in the articles [88-96] is that selection of a general practitioner is a deliberate choice process, the extent of information available about the practice is a factor, and that customer satisfaction is important. Direct accessibility to health services, as compared to the gate-keeping role of GPs, was important in one study [94] which included 14 European countries.

The health seeking behaviour of patients has also been revealed in articles about changes in the market and GP response to providing services in regard to consumer choice. Twelve articles [96-107] explore issues in the supply of general practitioner services to population groups in different types of markets in different countries. A common theme is that the supply of general practitioner services is changing in response to changes in the market, which may be as a result of government intervention, consumer preferences or other factors. Three articles [104-106] describe the growth and characteristics of retail health clinics in the USA. The articles note that the retail clinics, whether they are catering for ignored markets or new innovations, are siphoning patient care visits away from primary care general practitioner because of their attention to quick access, affordable prices and consumer-friendly approaches. Typically, the retail clinics cater for populations within a 5 minute driving distance, much less than the assumed driving distances in established GIS catchment models such as the 2SFCA method. The articles note that retail clinics may disrupt a number of aspects of existing primary care, including the range of services as well as market share of catchments. Another article [101] from the USA identifies that primary care general practitioners need to adopt different strategies according to the market in which they operate. The article notes the changes in market dynamics since 2004, which include the changes in employment contractual arrangements, business models and the percentage of primary care general practitioner employed in large delivery health systems (changed from 20 per cent in 2004 to over 40 per cent in 2012). The article identifies three different types of markets and advocates that general practitioners need to consider broader system goals in order to identify the right primary care strategy.

In summary, the consumer voice and choice in research studies as to their perceptions of GP catchments within the context of the relevant health market is an important area for study. The above articles highlight that consumers do not necessarily choose the nearest general practice to their place of residence when choice is available in the market. The relationship of GP catchments within the relevant health market and consumer expectations of what they expect of primary health care services needs more critical analysis.

#### Studies of GP catchments in relation to issues of primary health policy and regulation

There were twenty papers [98-100,108-124] that explored the role of government in regulating or changing the primary care health service. A common theme of the articles relates to the role of government intervention as to whether it is seen as a desirable legal regulatory control or a distortion of market forces in the allocation of health resources. For example, in England, the articles [110-114] explore the ideology of the government in creating local competitive markets comprising state, private and not-for-profit providers (with an independent regulator), based on the premise that a new primary care market should break the general practitioners’ monopoly of the provision of primary care and increase choice for consumers. Some of the articles note that there is significant uncertainty as to whether the policy will deliver desired objectives. Since 2012, government legislation has resulted in further changes in general practitioner markets [114].

Similar themes are covered by articles from Norway, Holland, and Germany and in articles in the USA [115,116,119]. Gingrich [107] synthesizes what is known about markets within the welfare states of Europe and England. Markets, according to Gingrich in an analysis of social services including primary health care, schools and long term care, vary across policy areas and countries. Government policy therefore, Gingrich summarises, has a strong influence on the types of markets that exist and evolve in primary health care settings. Policy changes alter who has control over production (ie. state/users/producers) and how resources are allocated and accessed, resulting in different welfare outcomes within these markets and general practitioners ’ share of the market.

The relationship between the concept of a total market for GP services and the catchments of individual GPs or general practices within the overall market requires further study. Several large scale studies, such as those of Busato [100] in Switzerland and McRae [98] in Australia have explored aspects of the GP supply of services within the overall market and the implications for patients wishing to access a GP. Both studies have implications for governments wishing to control the supply of GP services in a financial environment of cost cutting measures. For example, McRae showed if the Medicare rebate increases, GPs do not need to see as many patients to achieve the desired level of income. Busato [100] argues that freezing the number of general practitioners may have only a very limited effect on the associated resource utilisation.

In summary, studies from a number of countries show that changes in market dynamics therefore affect the supply of general practitioner services to consumers in ways that have unintended consequences. Government policy and regulation changes to the health market at the macro level do not necessarily translate into the uniform desired equity health outcomes for the population at the local level, where private general practices will adjust the supply of services, and thus the size and nature of their catchments, based on maintaining or enhancing their profitability.

## Discussion

This review has highlighted a wide range of diversity in the literature as to how GP catchments can be described, the indicators and measures used to frame the scale of catchments, as well as policy perspectives of different governments as to the role of general practice in provision of primary health care services in the relevant health market. Given the current health reform debate in many countries with a focus on reduced expenditure, there is a pressing need to have accurate empirical data on how and where patients access the services of general practitioners. This may need a re-conceptualisation as to what is understood to be a GP catchment.

There is a need to reconceptualise the concept of place based perspectives and explore the role of time geography Any model that assumes people should be structured only around the residential neighbourhood, treating individuals as if they were static and tied to their residential neighbourhood has significant limitations when considering the behaviours of people in developed countries. It should be more appropriate to integrate people’s activity space (e.g. the space about which they travel or move about in the course of their daily activities) when studying the determinants of health inequalities. When these factors are considered, an analysis of catchment patterns of general practitioners should be considered as dynamic and multi-perspective. Studies need to consider how people locate in place; how they move through space in their communities of interest and studies should also consider how they relate to people in space and place. In summary, patient access to general practice health care services should be considered from a range of locational concepts, and not necessarily constrained by their place of residence. Current assumptions about gaps in service provision need to be re-evaluated.

With regard to GP catchment models, the absence of real world data has been identified as a significant weakness of the GIS modelling. Other models, which use real world data such as that developed by Lewis and Longley to map actual GP catchment patterns are extremely important and provide opportunities for health geography to make significant contributions to informing reform on primary health care.

In terms of the inter-relationship between catchments and primary health care issues, the literature identified the role of governments as playing an important role in the regulation of the primary health markets and therefore exerting a strong influence on the types of markets that exist and evolve in primary health care settings. For example, it was identified in England that successive governments have introduced a range of regulations and systems of health delivery to break the monopoly of general practitioners in the primary health market. As noted in the introduction, in the USA, there is widespread debate as to the role of geography in priority setting for future health reform. Thus there is widespread uncertainty as to the effects of these programs on existing markets and catchments of general practice. There is a lack of evidence that such programs will universally provide better access for patients, better health outcomes or reduce health inequalities. It is possible that the effect of government policy will have the perverse result of causing poorer access for patients to GP services. This is an area of research where there is a gap and where there is a need for more research. Promising areas for future research should investigate the spatial and aspatial components of GP catchments, especially at the micro level, and model the effects of changes in the health policy settings of governments on equitable access to services by patients.

### Limitations

There are a number of limitations to this review including the challenge of containing the review study to the catchments of private sector general practice. There were over 1,300 articles identified in the initial search, and from this large cohort, the exclusion criteria required the elimination of many articles in order to meet the logistical aims of the review. Inevitably, it is possible that some articles which were excluded should have been included. Inevitably, if journal articles from more countries were included, the quality of the review could have been enhanced.

## Conclusions

This review has examined studies in relation to GP catchments from a range of developed countries. Patient access to general practice health care services should be considered from a range of locational concepts, and not necessarily constrained by their place of residence. An analysis of catchment patterns of general practitioners should be considered as dynamic and multi-perspective. Geographic information systems provide opportunities to contribute valuable methodologies to study these relationships. However, researchers acknowledge that a conceptual framework for the analysis of GP catchments requires access to real world data. The relationship of GP catchments to the health seeking behaviour of consumers and increasingly diversified markets for health services is complex. Recent studies have shown promising developments in the use of real world data, especially from studies in the UK. Understanding the catchment profiles of individual GP surgeries is important if governments are serious about patient choice being a key part of proposed primary health reforms.

## Abbreviations

2SFCA: Two step floating catchment area method; PHA: Practice Health Atlas.

## Competing interests

The author declares that he has no competing interests.

## Authors’ contribution

DA designed and formulated the research topic, searched the literatures, analyzed the data and wrote the manuscript. The author read and approved the final manuscript.
